# Impact of the Timing of Rocuronium Injection after Propofol Administration on Temporal Summation of Pain in Gynecologic Laparoscopic Surgery: A Prospective and Controlled Study

**DOI:** 10.1155/2020/6642460

**Published:** 2020-12-09

**Authors:** Jaehak Jung, Byoungryun Kim, Seong Nam Park, Jiheui Lee, Insung Choi, Myeong Jong Lee, Hyeonbin Yim, Cheol Lee, JuHwan Lee

**Affiliations:** ^1^Department of Obstetrics & Gynecology, Wonkwang University School of Medicine, 895 Muwang-ro, Iksan, Jeonllabuk-do 54538, Republic of Korea; ^2^Department of Anesthesiology and Pain Medicine, Korea Cancer Center Hospital, 75 Nowon-ro, Nowon-gu, Seoul 01812, Republic of Korea; ^3^Department of Anesthesiology and Pain Medicine, Chungju Hospital, Konkuk University School of Medicine, Chungju, Republic of Korea; ^4^Department of Anesthesiology and Pain Medicine, Wonkwang University School of Medicine, 895 Muwang-ro, Iksan, Jeonllabuk-do 54538, Republic of Korea

## Abstract

**Purpose:**

Temporal summation of pain, which is defined as the perception of greater pain evoked by repetitive painful stimuli, varies among individuals. This study aimed at determining the impact of the timing of rocuronium after induction with propofol on the temporal summation of pain.

**Methods:**

One hundred patients aged 19–60 years underwent gynecologic laparoscopic surgery. Patients were randomly assigned to one of the two groups: group PRi received immediate injections of rocuronium after propofol administration and group PRd received rocuronium injections when the bispectral index score (BIS) decreased to <60 after propofol administration. The grade of rocuronium-induced withdrawal movement (RIWM) according to the timing of propofol injection, the incidence and severity of propofol injection pain (PIP), rescue analgesics, visual analog scale (VAS) score after surgery for postoperative pain, patient-controlled analgesia (PCA) opioid consumption, association between PIP and the grade of RIWM, and associations between PIP, the grade of RIWM, and postoperative pain outcomes were measured.

**Results:**

The differences between the incidence and severity of PIP in the two groups were not significant. The grade of the RIWM in the PRd group was significantly reduced compared with the PRi group. Rescue analgesics, severity for postoperative pain, and PCA opioid consumption were not significant. Correlations between the incidence and severity of PIP and the grade of RIWM were weakly negative. Correlations between the grade of RIWM and pain outcomes were moderately positive, but correlations between the severity for PIP and the postoperative pain outcomes were negligible.

**Conclusion:**

The timing of rocuronium administration after propofol injection played a role in reducing RIWM. The grade of RIWM was significantly related to pain outcomes compared with the severity of PIP. Therefore, delayed rocuronium injection after induction with propofol reduced temporal summation of pain.

## 1. Introduction

Temporal summation of pain is an increase in pain perception in response to repeated exposure to painful stimuli. Individuals with increased pain processing and/or reduced pain-modulatory capabilities are regarded as pronociceptive, whereas those with reduced pain processing capacity are characterized as antinociceptive. The perceptions of a noxious stimulus may differ among individuals [1].

Propofol and rocuronium, which are used as anesthetic induction agents during general anesthesia, are often associated with pain or withdrawal movement (25%–100% for propofol and 22%–84% for rocuronium) in most patients [2, 3]. The characteristics of pain associated with propofol and rocuronium are inconsistent. One study reported that the characteristics of pain associated with the two anesthetic agents are similar; the pain occurs during administration, its duration is short, and its intensity decreases with subsequent injection [4]. However, another study reported that the time relationship and the nature of the pain or withdrawal movements associated with rocuronium are dissimilar from those associated with propofol [5]. Several methods have been attempted to reduce the frequency of pain or withdrawal movement after injections of the two drugs [6].

Several studies have reported the incidence or the severity of injection pain or withdrawal movement induced by the two anesthetic agents individually [2–8]. A few studies have reported the relationship between anesthetic depth using BIS and injection pain or withdrawal movement induced by the two anesthetic agents individually [7, 8]. However, the results of these studies were inconsistent. Few studies have reported the characteristics of the pain caused by individual anesthetic agents administered consecutively and the effect of the timing of subsequent anesthetic agent administration on pain/withdrawal movement.

We hypothesized that the timing of rocuronium administration after propofol injection may affect the rocuronium-induced withdrawal movement (RIWM), which was termed as the temporal summation of pain in this study. The timing of rocuronium administration after propofol injection was as follows: rocuronium was administered immediately after propofol injection or when the BIS decreased to less than 60 after propofol administration. Therefore, we investigated the frequency and severity of propofol injection pain (PIP), RIWM, and the associations between PIP, grade of RIWM, and postoperative pain outcomes (severity using VAS for pain at 1, 24, and 48 h after surgery and total opioid consumption for 48 h after surgery).

## 2. Materials and Methods

### 2.1. Study Design

Ethical approval for this prospective, randomized, and controlled study (registration no. 2020-04-033-002) was provided by the Wonkwang University Hospital Institutional Review Board (IRB) in April 2020. Written informed consent was obtained from all participants. The study was performed at the University Hospital from May 2020 to October 2020. Patients who were scheduled for laparoscopic gynecological surgery were enrolled in this study. The trial was registered at clinicaltrials.gov (NCT04547608) (see https://clinicaltrials.gov/ct2/show/NCT04547608).

### 2.2. Participants

The following patients were excluded: menopausal patients to exclude the hormonal effect on pain [9], those with muscular, cardiovascular, hepatic, or kidney disorders; those with a history of use of medications that could interfere with muscle relaxants; those with difficult venous access on the forearm; those with known propofol or rocuronium allergies; those with chronic pain; those who were pregnant; and those who had received analgesics or sedatives within the previous 24 hours. A total of 100 patients who were scheduled for gynecologic laparoscopic surgery, aged 19–60 years and classified as class I or II according to the American Society of Anesthesiologists (ASA), were enrolled in this study.

### 2.3. Randomization and Procedure

Randomization was performed using Stata 9.0 (StataCorp, College Station, TX, USA) statistical software. The patients were stratified using a 1 : 1 allocation and random block sizes of 4. All patients were assigned using simple randomization procedures (computerized random numbers) to 1 of 2 treatment groups: (a) group PRi (*n* = 50), which received an immediate injection of rocuronium after propofol administration, and (b) group PRd (*n* = 50), which received rocuronium injection when BIS decreased to less than 60 after propofol administration.

All patients were blinded to the group they were allocated to. All anesthetic procedures were performed by two attending anesthesiologists. One attending anesthesiologist performed anesthesia induction according to the study protocol. The other attending anesthesiologist measured all outcomes throughout the perioperative period.

### 2.4. Anesthesia and Perioperative Care

Intravenous 18-gauge cannula insertion in the forearm was performed by nurses in the wards for all patients in the morning before surgery. None of the patients were premedicated. In the operating room, all patients were assessed using the BIS monitor, electrocardiography, noninvasive arterial blood pressure (BP) measurement, and pulse oximetry.

Anesthesia was induced using 2 mg/kg of 1% propofol over 15 s (when considering arm brain circulation time (15–20 s)), and 0.6 mg/kg of 1% rocuronium was injected over 10 s.

An attending anesthesiologist observed patient movement during and after propofol and rocuronium administration. The patients were assessed using the VAS, with the scores ranging from 0 to 100 for pain severity after half-dose and full-dose propofol injection. If a patient could not respond to verbal questions after full-dose administration, the VAS score after half-dose administration was recorded.

The responses to rocuronium administration were graded on the following scale: 1 (none) = no response; 2 (mild) = movement at the wrist only; 3 (moderate) = movement involving the upper arm or shoulder; and 4 (severe) = movement in more than one extremity or a generalized response.

We adjusted the sevoflurane concentration using the mean arterial blood pressure (MBP) ± 20% and heart rate ± 20% and maintained BIS between 40 and 60. Neuromuscular blockade was reversed using pyridostigmine and glycopyrrolate when surgery was completed, and the train-of-four ratio (TOF) had returned to 25%. When patients started spontaneous breathing and BIS values reached 80, they were extubated.

A PCA pump containing fentanyl (800 *μ*g), ketorolac (150 mg), and ramosetron (0.6 mg) in 150 mL of saline was prepared to deliver a basal infusion of 2 mL/h and bolus doses of 0.5 mL, with a 15 min lockout period for postoperative analgesia. The pain severity after propofol injection and during the postoperative period was measured using a 100 mm linear VAS. The VAS score for pain during the postoperative period was measured during movement at 1, 24, and 48 h after surgery. When patients complained, pain corresponding to 50 mm or more on the VAS was treated with intravenous 100 *μ*g fentanyl. Ketorolac 30 mg was administered if the pain score was less than 40 mm on the VAS or the patient asked for analgesia.

### 2.5. Outcome Measures

The primary outcome was the grade of withdrawal movement induced by rocuronium administration according to the timing of propofol injection. The secondary outcomes included the incidence and severity of PIP, rescue analgesics, VAS score at 1, 24, and 48 h after surgery for postoperative pain, PCA opioid consumption at 24 and 48 h, the association between PIP and the grade of RIWM, and the associations between PIP, the grade of RIWM, and postoperative pain outcomes.

### 2.6. Sample Size and Statistical Analysis

The sample size was calculated using PASS 2008 (NCSS, LLC. Kaysville, Utah, USA). A preliminary investigation showed that the proportions of patients with no withdrawal movement induced by rocuronium administration after propofol injection in the two treatment groups were 0.312 and 0.590, respectively. Thus, a sample size of 47 patients per group would enable the detection of a significant difference with a power of 80% and an *α*-coefficient of 0.05. The final sample size in this study was 50 patients per group, after adjustment for a 5% dropout rate. SPSS version 18.0 (SPSS Inc., Chicago, IL, USA) was used for statistical analysis. The data are presented as mean ± SD or number (%) of patients. The groups were compared using the independent *t*-test or the Mann–Whitney *U* test for continuous variables depending on the normality of their distributions and the *χ*2 test or Fisher's exact test for categorical variables, as appropriate. The correlation between parameters was analyzed using Kendall's tau-b correlation test.

## 3. Results

**Figure 1:**
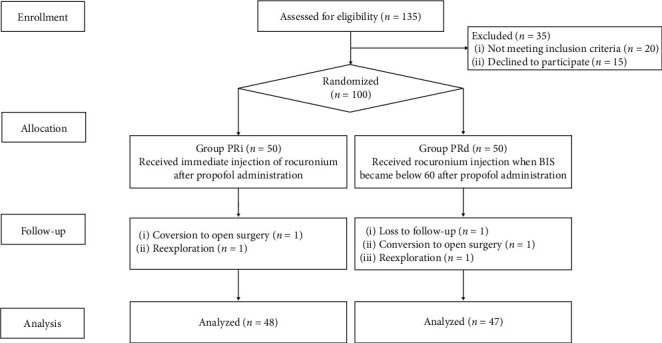
Consort flow diagram.

A total of 135 patients were assessed for eligibility, and 35 patients were excluded; 20 did not meet the inclusion criteria and 15 refused to participate. A hundred patients received medication after randomization. Five patients were withdrawn after enrolment due to conversion to open surgery, loss of follow-up, and reexploration for postoperative bleeding (Figure 1).

The differences between the ages, heights, weights, ASA classification, duration of anesthesia, duration of surgery, and the types of surgery of the two groups were not significant (Table 1).

The basal BIS and the BIS values of the two groups immediately after propofol injection were not significant. The time to obtain a BIS of <60 was significantly shorter in the PRi group than in the PRd group (*P*=0.00). The incidence and severity of PIP of the two groups assessed with VAS were not significant. The grade of the RIWM was significantly reduced in the PRd group than in the PRi group (*P*=0.019). The VAS scores for pain at 1, 24, and 48 h after surgery, PCA opioid consumption at 24 and 48 h after surgery, and total opioid consumption for 48 h after surgery of the two groups were not significantly different. Rescue analgesia (with ketorolac or fentanyl), tenderness/redness/hardness of the vein, tenderness and hardness of the vein, and recall (pain or respiratory difficulty) after surgery were not significantly different between the two groups (Table 2).

The correlations between the incidence (*r* = −0.25, *P*=0.012) and severity (*r* = −0.22, *P*=0.015) of PIP, assessed with VAS, and the grade of RIWM were weakly negative and significant (Table 3). The correlations between the grade of RIWM and the VAS score for pain at 1 h after surgery (*r* = 0.41, *P*=0.00), VAS score for pain at 24 h after surgery (*r* = 0.40, *P*=0.00), VAS scores for pain at 48 h after surgery (*r* = 0.34, *P*=0.04), or total opioid consumption for 48 h after surgery were moderately positive and significant, but the relationship between the VAS score of PIP and postoperative pain outcomes was negligible (Table 4).

## 4. Discussion

The main findings of our study demonstrated that the grade of RIWM was significantly lower in patients who received delayed rocuronium injection (BIS < 60) than in those who received immediate rocuronium injection after propofol administration. The incidence of RIWM and the incidence and severity of PIP, assessed with VAS, were consistent with those reported by previous studies [3–5].

The perceptions of pain may vary with age and sex, owing to biological or psychosocial mechanisms; they may even differ for individuals of the same age or sex because of their experience of pain [10–12]. This study included only female adults without menopause to reduce the effect of age and sex on pain outcomes. It is known that the two anesthetic agents used in this study have a very large range of incidence of injection pain or withdrawal movement. These may result from the abovementioned reasons. This study showed that two consecutive delayed pain stimuli could reduce pain perception. Therefore, this may be a method for reducing injury during induction due to gastric content reflux, pulmonary aspiration, and dislodging of the venous catheter following withdrawal movement [2, 13, 14].

The duration to obtain a BIS of <60 was significantly shorter in patients who received an immediate injection of rocuronium than in patients who received a delayed injection of rocuronium after propofol administration. This may have been an effect of electromyographic activity on the calculation of BIS [15]. This study showed that the incidence and severity of pain and the frequency of recall after propofol injection were similar to those reported in previous studies [16, 17]. Pain after propofol injection was mild, and the frequency of recall was low. The propofol injection pain was transient and acceptable.

This study showed that the correlations between the grade of RIWM and the VAS score for pain at 1 h after surgery, VAS score for pain at 24 h after surgery, VAS score for pain at 48 h after surgery, and total opioid consumption for 48 h after surgery were moderately positive and significant, but the relationship between the VAS score for PIP and postoperative pain outcomes was negligible. Although the two anesthetic agents caused pain or withdrawal movement, preoperative pain perception of rocuronium may be related to pain outcomes after surgery.

There were some limitations to this study. First, the sample may be small for evaluating the impact of the timing of rocuronium injection after propofol administration on the temporal summation of pain, although the sample size was determined by the proportion of patients with no withdrawal movement after rocuronium administration following propofol injection in the two treatment groups. A small sample may point the researcher to different directions during clinical decision making. Second, age and sex can affect pain perception. We performed gynecologic surgery in women without menopause to reduce selection bias. We did not consider the hormonal state of the women or the stages of their menstrual cycles, and this may have affected the results.

Finally, this study was not blinded to minimize observer bias. One attending anesthesiologist assessed the severity of injection pain after propofol administration, and the grading of RIWM was open, but other attending anesthesiologists who evaluated postoperative pain outcomes and complications were blinded to it.

In conclusion, the timing of rocuronium administration after propofol injection facilitated the reduction of RIWM. The grade of RIWM was significantly related to postoperative pain outcomes; the severity of PIP was not. Therefore, delayed rocuronium injection after induction with propofol reduced temporal summation of pain. Further studies are required to elucidate the temporal summation of pain for drugs used during anesthetic induction.

## Figures and Tables

**Table 1:** Patient demographic data.

	PRi group (*n* = 48)	PRd group (*n* = 47)	*P* value
Age (years)	38.4 ± 9.4	39.2 ± 8.7	0.67
Height (cm)	160.8 ± 3.4	160.8 ± 4.1	0.93
Weight (kg)	60.9 ± 7.5	61.4 ± 7.8	0.73
ASA (I/II)	16 (33.3)/32 (66.7)	14 (31.6)/33 (70.2)	0.71
Duration of anesthesia (min)	66.2 ± 28.6	67.1 ± 29.6	0.87
Duration of surgery (min)	95.6 ± 29.6	96.7 ± 29.9	0.86
*Type of surgery*			0.630
Laparoscopic myomectomy	9 (18.8)	7 (14.9)	—
Laparoscopic subtotal hysterectomy	7 (14.6)	9 (19.1)	—
Laparoscopic vaginal hysterectomy	19 (39.6)	13 (27.7)	—
Laparoscopic vaginal hysterectomy and lymph node dissection	6 (12.5)	7 (14.9)	—
Laparoscopic cystectomy	7 (14.6)	11 (23.4)	—

Values are expressed as mean ± SD or numbers (%).

**Table 2:** Perioperative data.

	PRi group (*n* = 48)	PRd group (*n* = 47)	*P* value
Basal BIS	95.1 ± 2.7	95.1 ± 2.8	0.99
BIS immediately after propofol injection	94.9 ± 2.2	95.0 ± 1.7	0.96
Average BIS less than 60	57.7 ± 1.7	57.7 ± 2.2	0.96
Time to get BIS less than 60 (sec)	28.4 ± 5.0	35.1 ± 4.9	0.00
The incidence of propofol injection pain	25 (52.1)	26 (55.3)	0.75
VAS for propofol injection pain	35.6 ± 12.6	36.9 ± 14.1	0.70
*The grade of rocuronium-induced withdrawal movement*	—	—	0.019
1 (no withdrawal)	15 (31.3)	26 (55.3)	—
2 (wrist withdrawal)	0 (0)	0 (0)	—
3 (arm only)	22 (45.8)	18 (38.3)	—
4 (generalized movement)	11 (22.9)	3 (6.4)	—
VAS for pain at 1 hr after surgery	43.5 ± 11.9	42.1 ± 9.8	0.53
VAS for pain at 24 hr after surgery	35.4 ± 12.0	32.6 ± 8.5	0.18
VAS for pain at 48 h after surgery	24.4 ± 10.1	21.7 ± 7.0	0.14
PCA opioid consumption at 24 h after surgery	52.7 ± 4.0	51.6 ± 3.8	0.14
PCA opioid consumption at 48 h after surgery	50.5 ± 2.8	49.8 ± 2.2	0.20
Total PCA opioid consumption for 48 h after surgery	103.1 ± 6.3	100.3 ± 9.5	0.10
*Rescue analgesics*			0.30
Ketorolac	19 (39.6)	25 (53.2)	—
Fentanyl	26 (54.2)	18 (38.3)	—
*Complications on vein*			
Tenderness	3 (6.3)	1 (2.1)	0.32
Redness	4 (6.3)	2 (4.3)	0.41
Hardness	1 (2.1)	0 (0)	0.32
Tenderness and hardness	1 (2.1)	0 (0)	0.32
*Recall*			
Pain/respiratory difficulty	5 (10.4)/0 (0)	4 (8.5)/0 (0)	0.75

Values are expressed as mean ± SD or numbers (%). PCA, patient-controlled analgesia; VAS, visual analog scale.

**Table 3:** The correlation between propofol injection pain and the grade of rocuronium-induced withdrawal movement.

	The grade of rocuronium-induced withdrawal movement
The incidence of propofol injection pain	*r* = −0.25
	*P*=0.012

VAS for propofol injection pain	*r* = −0.22
	*P*=0.015

VAS: visual analog scale.

**Table 4:** The correlations between propofol injection pain, the grade of rocuronium-induced withdrawal movement, and pain outcomes.

	VAS score for pain at 1 h after surgery	VAS score for pain at 24 h after surgery	VAS score for pain at 48 h after surgery	Total opioid consumption for 48 h after surgery
The incidence of propofol injection pain	*r* = −0.23	*r* = −0.23	*r* = −0.09	*r* = −0.23
	*P*=0.013	*P*=0.016	*P*=0.36	*P*=0.00

VAS for propofol injection pain	*r* = −0.18	*r* = −0.17	*r* = −0.053	*r* = −0.18
	*P*=0.38	*P*=0.04	*P*=0.55	*P*=0.03

The grade of rocuronium-induced withdrawal movement	*r* = 0.41	*r* = 0.40	*r* = 0.34	*r* = 0.46
	*P*=0.00	*P*=0.00	*P*=0.04	*P*=0.00

VAS: visual analog scale.

## Data Availability

The data used to support the findings of this study are available from the corresponding author upon request.
